# Simultaneous Hypoxia and Low Extracellular pH Suppress Overall Metabolic Rate and Protein Synthesis In Vitro

**DOI:** 10.1371/journal.pone.0134955

**Published:** 2015-08-14

**Authors:** Brita Singers Sørensen, Morten Busk, Jens Overgaard, Michael R. Horsman, Jan Alsner

**Affiliations:** Department of Experimental Clinical Oncology, Aarhus University Hospital, Aarhus, Denmark; University of Oxford, UNITED KINGDOM

## Abstract

**Background:**

The tumor microenvironment is characterized by regions of hypoxia and acidosis which are linked to poor prognosis. This occurs due to an aberrant vasculature as well as high rates of glycolysis and lactate production in tumor cells even in the presence of oxygen (the Warburg effect), which weakens the spatial linkage between hypoxia and acidosis.

**Methods:**

Five different human squamous cell carcinoma cell lines (SiHa, FaDu_DD_, UTSCC5, UTSCC14 and UTSCC15) were treated with hypoxia, acidosis (pH 6.3), or a combination, and gene expression analyzed using microarray. SiHa and FaDu_DD_ were chosen for further characterization of cell energetics and protein synthesis. Total cellular ATP turnover and relative glycolytic dependency was determined by simultaneous measurements of oxygen consumption and lactate synthesis rates and total protein synthesis was determined by autoradiographic quantification of the incorporation of ^35^S-labelled methionine and cysteine into protein.

**Results:**

Microarray analysis allowed differentiation between genes induced at low oxygen only at normal extracellular pH (pH_e_), genes induced at low oxygen at both normal and low pH_e_, and genes induced at low pH_e_ independent of oxygen concentration. Several genes were found to be upregulated by acidosis independent of oxygenation. Acidosis resulted in a more wide-scale change in gene expression profiles than hypoxia including upregulation of genes involved in the translation process, for example Eukaryotic translation initiation factor 4A, isoform 2 (EIF4A2), and Ribosomal protein L37 (RPL37). Acidosis suppressed overall ATP turnover and protein synthesis by 50%. Protein synthesis, but not total ATP production, was also suppressed under hypoxic conditions. A dramatic decrease in ATP turnover (SiHa) and protein synthesis (both cell lines) was observed when hypoxia and low pH_e_ were combined.

**Conclusions:**

We demonstrate here that the influence of hypoxia and acidosis causes different responses, both in gene expression and in de novo protein synthesis, depending on whether the two factors induced alone or overlapping, and as such it is important for in vivo studies to take this into account.

## Introduction

Solid malignant tumors are characterized by an inadequate vascular system, which can give rise to microregional areas deprived in nutrients and oxygen and enriched with acidic waste products [1,2]. This leads to a tumor microenvironment characterized by hypoxia and low pH. The physiological stresses of hypoxia and acidosis have shown to lead to a more malignant tumor phenotype linked with metastasis, invasion, and treatment resistance [3–6].

Acidosis in solid tumors is mainly caused by lactic acid accumulation. Hypoxia stimulates glycolysis and the formation of lactic acid in order to compensate for reduced mitochondrial ATP production in a process resembling the influence oxygen exerts on fermentation in yeast, referred to as the Pasteur effect after its discoverer Louis Pasteur [7]. Since tumor cells maintain a high rate of glycolysis even in the presence of oxygen, a phenomenon known as aerobic glycolysis or the Warburg effect [8], significant disparities in the spatial and temporal distribution of areas with low pO_2_ and low extracellular pH (pH_e_) in tumors exist [9,10]. Studies have shown microregional tumor pH_e_ levels in the range of 6.15 to 7.5 [2,9], and a lack of correlation between pH and oxygen levels [9]. Acidosis has previously demonstrated to influence cellular responses, such as stimulating autophagy [11], and to effect gene expression [12–14]. Hypoxia induces multiple responses, including elevated glycolysis, reduction of cell proliferation, and stimulation of angiogenesis and erythropoiesis. These effects are orchestrated by the hypoxia inducible factor (HIF), which is the main hypoxic switch [15].

Studies addressing hypoxia and acidosis have demonstrated an interaction of these two factors. Mekhail et al. showed that VHL, the protein that controls degradation of HIF-1α, was affected by low pH_e_ (6.3), in that acidosis triggered a nucleolar sequestration of VHL, and thereby neutralizing the HIF-1 degrading function [16]. Previous studies have shown a different cellular response in term of gene expression and of DNA repair following hypoxia and acidosis in combination compared to either hypoxia or acidosis alone [12]. Furthermore, studies have demonstrated that extracellular pH influences hypoxia related gene expression to a large extent [13,14], with low pH_e_ suppressing the hypoxia induced up-regulation of gene expression in a number of genes, especially CA9. A recent study has observed additive effects of hypoxia, acidosis and interstitial fluid pressure on a range of factors in tumor cell biology [17]. These findings highlight the importance of considering the two factors, hypoxia and acidosis, both separately and in combination. This is both in order to obtain solid biological markers for both conditions, but also to understand their distinct contributions to tumor phenotype. Clearly, being able to accurately distinguish the relevant microenvironmental factors and unravel how these factors interact or act independently will help us to understand the biology of the tumor microenvironment.

We have previously published an *in vitro* study where five carcinoma cell lines (SiHa, FaDu_DD,_ UTSCC5, UTSCC14 and UTSCC15) were exposed to different micro-environmental factors followed by gene expression analysis to identify a cell-line and pH_e_ independent hypoxia profile [18]. In the present study, we have used the same dataset to identify genes affected by acidosis.

## Methods and Materials

### 1. Cell culture and hypoxia treatment

The cell culture and hypoxia treatment has previously been described in [18]. The human uterine cervix squamous cell carcinoma (SiHa) cell line was obtained from the American Type Culture Collection (ATCC, Rockville, MD). The head and neck squamous cell carcinomas FaDu_DD_, a subline of FaDu (an undifferentiated hypopharygeal SCC), UtScc5, UtScc14, UTSCC15 and UTSCC33 (established by Dr. Reidar Grenman, University of Turku, Finland, and previously described in [19]), were obtained from Dr. Michael Baumann. Cells were cultured in Eagle’s minimal essential medium with glutaMAX I and Earle's balanced salt solution (Gibco) containing 1.5g/L sodium bicarbonate, 0.1 mM nonessential amino acids, 1.0 mM sodium pyruvate, 10% fetal calf serum, 100,000 U/L penicillin, and 100 mg/L streptomycin (SiHa), or in Dulbeccos modified Eagle medium with glutaMAX I containing 1.5g/L sodium bicarbonate, 2% HEPES, 0.1 mM nonessential amino acids, 1.0 mM sodium pyruvate, 10% fetal calf serum, 100,000 U/L penicillin, and 100 mg/L streptomycin (FaDu_DD_, UTSCC5, UTSCC14 and UTSCC15), with 5% CO_2_ in a well humidified incubator. Prior to experiments, 2x10^5^ cells were transfered into 60mm glass Petri dishes three days before start of experiment. Cells were in the log-phase of growth at the time of experiment. Hypoxia was induced by continually gassing the cells in an airtight chamber for 24 hours with 21, 5, 1, 0.1, 0.01 or 0% oxygen, supplemented with 5% CO_2_ and nitrogen as balance, at 37°C. To induce acidosis, pH of the medium (without sodium bicarbonate) was buffered with 20mM tris(hydroxymethyl)aminomethane (TRIS) base (Sigma), 20mM 2-(N-morpholino)ethanesulfonic acid (MES) (Sigma) and 0.52 g/l NaHCO_3_ (Sigma) and titrated to 6.3 or 7.5. The pH adjusted media was applied immediately before the induction of hypoxia. Cell mortality under all conditions was below 5%. As a control of the potential influence of the buffer system on gene expression, an additional experiment with conventional sodium bicarbonate buffered cell media with added lactate, and the pH titrated to 7.3, 7.15 and 6.7, was performed.

### 2. RNA extraction and oligonucleotide microarray analysis

The RNA extraction and microarray analysis has previously been described in [18]. Immediately after removal from the airtight chamber, media was removed and 350 μl RTL buffer (Qiagen) containing 10 ml/l β-mercaptoethanol (Merch, Germany) was added to the Petri dishes. Cells were homogenized in a QiaShredder filter (Qiagen) at 10,000 g for 2 minutes. RNA was extracted using the RNeasy Mini Kit (Qiagen) according to the manufactures instructions. RNA eluted in RNase free water was quantified using a spectrophotometer (SmartSpec Plus, Biorad). To analyse gene expression across the whole genome, the samples was analyzed on the Human Genome U133 Plus 2.0 Array (AROS Applied Biotechnology, Aarhus, Denmark). All array data was MAS 5.0 global scaled using Affymetrix Expression Console 3.1 Software package. For the gene clustering analysis, data, including probesets with known unique gene ID, was analyzed with Gene Cluster 2.11 (Michael Eisen, rana.lbl.gov/EisenSoftware.htm) using Pearsons correlation and complete linkage, Java Treeview (ver 1.1.3) and SAM (ver 3.02).

All array data is available at GEO, NCBI (Accesion number GSE70051).

Data sets were subjected to enrichment analysis (Geneontology.org, Analysis type: PANTHER Overrepresentation Test (release 20150430), GO Ontology database Released 2015-06-06).

### 3. Reverse transcription and qPCR

The qPCR method has previously been described in [18]. cDNA was generated using the High Capacity cDNA Archive kit (Applied Biosystems; ABI) according to the manufacturer’s instructions. Total RNA (2 μg) was reverse transcribed using random hexamer primers. To detect transcripts of interest, TaqMan Gene Expression assay (ABI) was used for EIF4A2 (Hs01115195_g1) and JOSD3(Hs00936351_g1). For each reaction, cDNA (corresponding to 20ng RNA), 1x assay mix and 1x Taqman Universal PCR mastermix (ABI) in a total of 25 μl was mixed. Reactions were performed on an ABI Prism 7000 Sequence Detector (ABI) (40 cycles). All reactions were performed in triplicate. Results were normalized to the three reference genes TFRC (4333770F), GUSB (4333767F), and HPRT1 (4333768T). These reference genes was selected on the basis of a TaqMan Human Endogenous control plate (ABI) on which cDNA from the different treatments was analyzed against 12 common reference genes [14], and the geNORM Visual Basic application available in RealTime Statminer (Intergromics). Data analysis was performed using the RealTime Statminer software (Intergromics), and the Comparative C_T_ method. To test for whether genes were significantly upregulated under hypoxia and acidosis relatively to the control conditions (Atmospheric air + pH 7.5), a two-tailed T-test was used. To correct for multiple comparisons, Bonferroni-Holm corrections was applied.

### 4. Pulse-labelling assay and immunoblotting

To determine whether cells modified the level of protein synthesis during the period of anoxia and/or acidosis, cell media (pH 7.5 and pH 6.3) was supplemented with 0.1 mCi EasyTag EXPRESS35S Protein Labeling Mix (Perkin Elmer). The media was applied to the cells immediately before the induction of anoxia/acidosis. The cells were incubated in either atmospheric air (controls) or 0% oxygen supplemented with 5% CO_2_ for 24 hours at 37°C. Cells were immediately after treatment rinsed in 2x D-PBS, and lysed in 400μl lysis buffer (Complete Lysis-M (Roche)). Protein quantity was quantified using DC protein assay (Biorad). Equal quantities of protein were resolved on 10–20% Tris-Glycine gels (Invitrogen). Quantitative autoradiography analysis of gel samples was performed using a Bas5000 laser-excited image analyzer (Fuji Film, Tokyo, Japan) and quantitated using the ImageGauge 4.0 software. As loading control, an identical gel were blotted onto nitrocellulose membranes (Invitrogen), which were incubated with antibody against beta actin (Abcam ab8226, 1:5000 dilution), followed by incubation with peroxidase-conjugated secondary antibody (anti-rabbit, DAKO, dilution 1:5000). Protein signal was detected using the Super Signal West Pico kit (Pierce). To test for significant differences in protein levels under hypoxia and acidosis relatively to the control conditions (Atmospheric air + pH 7.5), a two-tailed T-test was used.

### 5. In vitro oxygen consumption and lactate formation rates

Rates of oxygen consumption (OCR) and lactate production (LPR) were measured in attached cells approximately 12h (i.e., midway in the period over which protein synthesis rate was measured) after initiation of exposure to the conditions of interest. In experiments involving cells exposed to atmospheric conditions at a normal (7.5) or low (6.3) medium pH, OCR and LPR were measured simultaneously in a custom built cellular respirometer (described in detail in [20]). In anoxic experiments, cells in medium with a normal or low pH were placed in air-tight boxes and flushed with a gas mixture of 5% CO_2_ and 95% N_2_. LPR was calculated from the difference in [lactate] in two medium samples. Following media sampling, cells were counted and OCR and LPR were expressed as fmol/min/cell. Since a relatively high cell density is required for accurate measurements of OCR in the cellular respirometer, confluent cells were used for characterization of the basal energy budget under both normoxic and anoxic conditions. A simplified basal energy budget consists of a) ATP formation linked to mitochondrial oxidation of glucose (includes also the ATP generated in glycolysis if glucose is oxidized) and b) mitochondrial-independent ATP formation linked to the formation of lactate. From these two ATP generating sources, the relative contribution from glycolysis to the total ATP energy budget can be calculated as: LPR/(LPR + 4.5×OCR). This formula assumes that (a) lactate originates from sources other than endogenous glycogen resulting in the production of 1 mole ATP per 1 mole of lactate accumulated and (b) passive mitochondrial proton leakiness (causing no ATP-synthesis) dissipates 25% of the energy stored in the proton gradient, leading to the generation of only ~4.5 mole of ATP per mole O_2_ consumed. Proton leakiness is now a generally accepted phenomenon (for a review see [21]). It may vary between different organs and cell types but its exact magnitude will not affect our conclusions, since we are interested in quantifying relative differences between the different incubation conditions for a given cell line.

To test for significant differences in lactate production rate and oxygen consumption rate, a two-tailed T-test was used, comparing high vs low pH in oxygenated and high vs low pH in anoxic conditions, respectively.

## Results

### 1. Gene expression response in cells treated with hypoxia and acidosis

**Fig 1:**
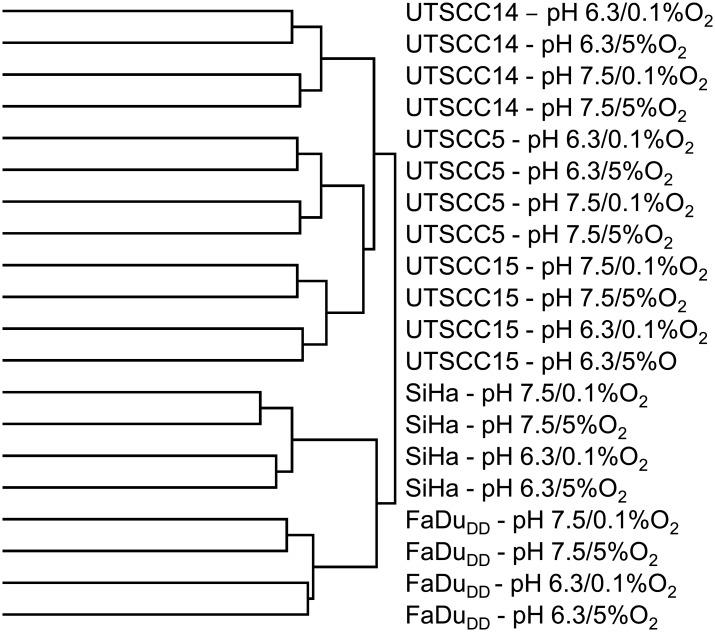
Effect of tumor microenvironmental factors on gene expression. Unsupervised hierarchical clustering (median centred genes, complete linkage) of gene expression data from SiHa, FaDu_DD_, UTSCC5, UTSCC14 and UTSCC15 cells treated with combinations of oxygen concentrations (0.1%O_2_ and 5%O_2_) and pH_e_ (6.3 and 7.5).

A panel of human squamous cell carcinoma cell lines (SiHa, FaDu_DD,_ UTSCC5, UTSCC14 and UTSCC15) was treated with different grades of hypoxia and pH_e_, and gene expression was analyzed by Affymetrix Gene Expression Microarray. Data for all cell lines was analyzed by unsupervised hierarchical clustering (Fig 1). This demonstrated that the genes separated into main groups representing the five cell lines. Within all cell line, samples then clustered into high and low pH, showing that acidosis affected cellular gene expression profiles more profoundly than hypoxia.

To specifically select for pH regulated genes across more cell lines, data were analyzed to select genes based on different criteria. For genes to be considered as pH induced, expression had to be at least 2 fold upregulated at low pH under both hypoxic and normoxic conditions. In Table 1, the number of upregulated genes in the individual cell lines, and the biological processes the genes are involved in, are given. For each cell line, between 221 and 662 genes were more than 2 fold upregulated at low pH, whereas 107–289 genes were upregulated at low pH, both at normoxia and hypoxia.

**Table 1:** Number of upregulated genes in individual cell lines and top five of GO Biological Processes for each gene set.

Cell line	Number of genes upregulated at low pH	GO Biological Processes	Number of genes upregulated at low pH—independent of hypoxia	GO Biological Processes
**SiHa**	437	Cellular response to arsenic-containing substance	215	Response to hydrogen peroxide
		Response to arsenic-containing substance		Response to reactive oxygen species
		Positive regulation of smooth muscle cell proliferation		Response to oxidative stress
		Regulation of smooth muscle cell proliferation		Positive regulation of programmed cell death
		Negative regulation of cysteine-type endopeptidase activity involved in apoptotic process		Blood vessel development
**FaDu**	300	Myoblast differentiation	139	Regulation of signaling
		Negative regulation of protein serine/threonine kinase activity		Regulation of cell communication
		Reproductive system development		Response to organic substance
		Reproductive structure development		Regulation of signal transduction
		Monocarboxylic acid metabolic process		Organ development
**UTSCC5**	221	Response to arsenic-containing substance	107	Cellular response to organic substance
		Response to zinc ion		Response to organic substance
		Regulation of cysteine-type endopeptidase activity involved in apoptotic process		Signal transduction
		Cellular response to external stimulus		Single organism signaling
		Response to metal ion		Signaling
**UTSCC14**	662	Sequestering of metal ion	289	Regulation of extrinsic apoptotic signaling pathway via death domain receptors
		Type I interferon signaling pathway		Response to interferon-gamma
		Cellular response to type I interferon		Cytokine-mediated signaling pathway
		Response to type I interferon		Regulation of apoptotic signaling pathway
		Regulation of extrinsic apoptotic signaling pathway via death domain receptors		Cellular response to cytokine stimulus
**UTSCC15**	615	Antigen processing and presentation of exogenous peptide antigen via MHC class I, TAP-independent	286	Antigen processing and presentation of exogenous peptide antigen via MHC class I, TAP-independent
		Response to zinc ion		Type I interferon signaling pathway
		Type I interferon signaling pathway		Cellular response to type I interferon
		Cellular response to type I interferon		Response to type I interferon
		Response to type I interferon		Negative regulation of viral genome replication

**Fig 2:**
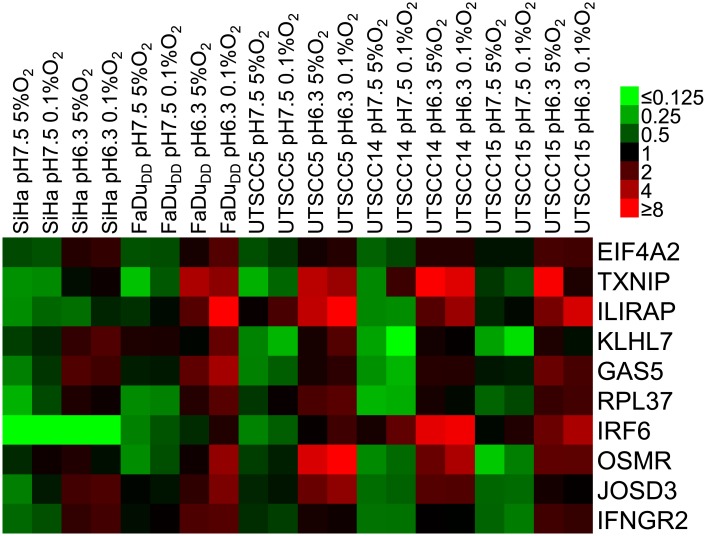
pH induced genes across cell lines. Expression (microarray data) of genes found to be more than 2 fold upregulated under low pH independent of hypoxia in 4 out of 5 cell lines (SiHa, FaDu_DD_, UTSCC5, UTSCC14 and UTSCC15). Data displayed is log transformed and median centered.

When comparing across the cell lines, 14 probesets, representing 10 different genes, were upregulated more than 2 fold under low pH independent of hypoxia in 4 out of 5 cell lines (Fig 2 and S3 Fig), with three of the genes being upregulated in all 5 cell lines (TXNIP, GAS5 and RPL37). The fold changes at different conditions is presented in S1 Table.

The included microarray data for FaDuDD and SiHa cells were part of a larger study, were the following conditions were investigated: 21, 5, 1, 0.1, 0.01 or 0% oxygen at pH 6.3 or 7.5 (S1 Fig). This data was analyzed with a multiclass Significance Analysis of Microarray (SAM), with the samples combined into four groups: “Normal oxygen, normal pH” (21%, pH 7.5; 5%, pH 7.5), “Low oxygen, normal pH” (0.1%, pH 7.5; 0%, pH 7.5;), “Normal oxygen, low pH” (21%, pH 6.3; 5%, pH 6.3), “Low oxygen, low pH” (0.1%, pH 6.3; 0%, pH 6.3). More details on this analysis is given in S1 Methods. The group of genes that was induced at low pH independent of oxygen concentration in this analysis (S2 Fig), included a subset of genes common between the two analysis (e.g. EIF4A2, JOSD3 and RPL37).

### 2. Expression levels of EIF4A2 and JOSD3

**Fig 3:**
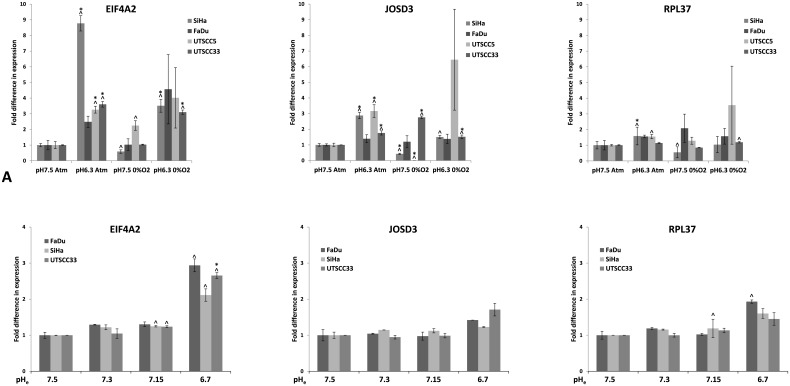
pH induction of identified genes. Relative levels of EIF4A2, JOSD3 and RPL37 mRNA measured by qPCR in SiHa, FaDu_DD_, UTSCC5 and UTSCC33 cells under different conditions. Levels are normalized to the control samples (pH 7.5, atmospheric oxygen level). Results are mean of three independent experiments (+/-SEM). (^) indicates p values <0.05 compared to the control level. (*) indicates p values which are significant different from the control levels, when correction for multiple comparisons is applied.

The expression of three of the genes from the analysis, EIF4A2, JOSD3 and RPL37 was further explored by qPCR at different oxygen concentrations, presenting the relative expression levels normalized to the control sample (21%O_2_ at pH 7.5), in four different HNSCC cell lines (SiHa, FaDu_DD_, UTSCC5 and UTSCC33) as shown in Fig 3A. 21%O_2_ and 0%O_2_ were chosen for this analysis, as they represent the most radical conditions. It can been seen that the expression of EIF4A2 increased significantly for three out of four cell lines at low pH with normal oxygen concentrations, and for two of the tested cell lines at low pH with 0% oxygen, relative to the control samples (cells at pH 7.5 at normal oxygen concentration). The expression of JOSD3 increased significantly for three out of four cell lines at low pH with normal oxygen concentrations. At pH 6.3, 0% O_2_ the expression of JOSD3 was only significantly increased in one out of four cell lines. A decrease in expression for two of the cell lines at pH 7.5, 0% O_2_, relative to the levels at pH 7.5, normal oxygen concentration was observed. Expression of RPL37 was only significantly increased in one out of four cell lines at pH 6.3, atm air.

In an additional experiment, the expression of EIF4A2, JOSD3 and RPL37 was tested in SiHa, FaDu_DD_ and UTSCC33 at normal oxygen concentration at a pH range from 7.5 to 6.7 (Fig 3B), which was obtained using a different buffering system and changing the lactate concentration. In these experiments EIF4A2 showed a significant increase at pH 6.7 for one of the three cell lines, whereas JOSD3 and RPL37 did not exhibit a significant increase in expression at pH 6.7, compared to expression levels at pH 7.5.

### 3. Effect of anoxia and acidosis on protein synthesis

**Fig 4:**
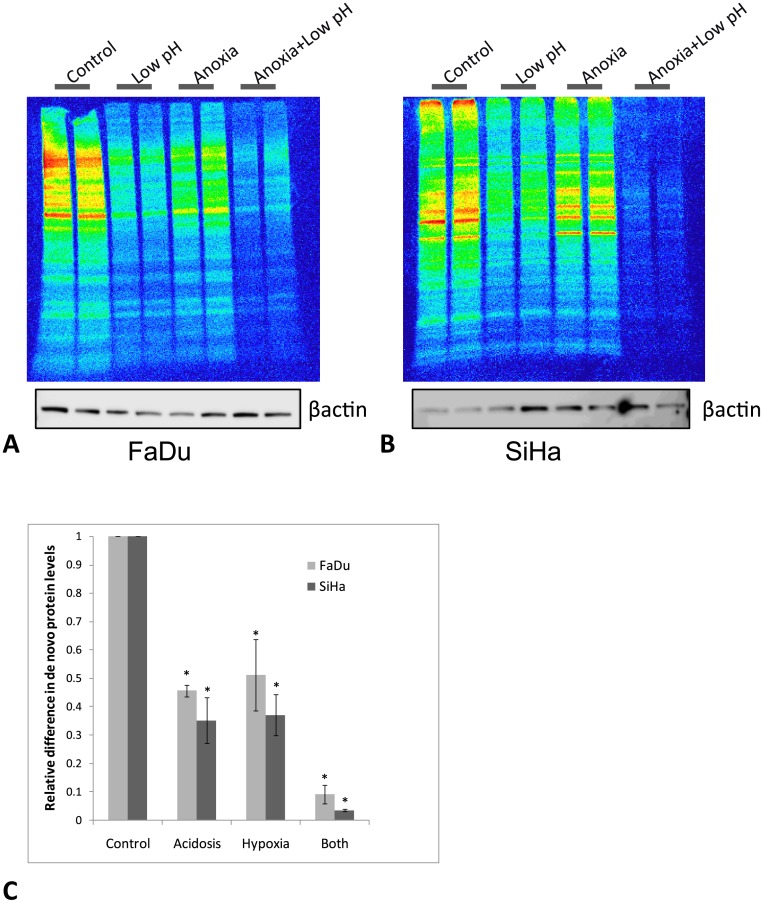
Pulse-labelling assay. To determine whether the cells modified their levels of protein synthesis, ^35^S-labelled methionine and cysteine was added during treatment, and de novo protein levels were determined by autoradiography. The gels shown in A (FaDu) and B (SiHa) are representative gels with equal amounts of total protein loaded (two lanes are loaded with each sample). βactin levels was measured on similar gels. C: Mean values from three independent experiments for SiHa and FaDu_DD_ cells (+/-SEM). (*) indicates p values <0.05 compared to the control level.

Several of the genes identified to be upregulated by low pH are involved in protein translation. We therefore wanted to see how a change in pH and/or oxygen concentration affected the level of protein synthesis. In a pulse labeling experiment using SiHa and FaDu_DD_ cells, ^35^S labeled amino acids were added in the cells growth media at the time of inducing acidosis and/or anoxia. The cells were incubated at these conditions for 24 hours and harvested. Equal levels of total protein were resolved on a polyacrylamide gel, and protein levels were determined by digital autoradiography. Fig 4 shows representative gels depicting the levels of protein synthesized during treatment of FaDu_DD_ cells. To ensure equal levels of total protein in the samples, β-actin levels were measured by immunoblots of identical gels. Three independent experiments for both cell lines have been conducted (additional gel pictures are shown in S4 Fig). All experiments showed a significant decrease in protein synthesis level (around 50–60%) when the cells had been treated with either low pH or anoxia, whereas a combination of the two factors in both cell lines led to a drastic decrease in the level of protein synthesized (>90% decrease).

### 4. Effect of anoxia and acidosis on ATP generation

**Fig 5:**
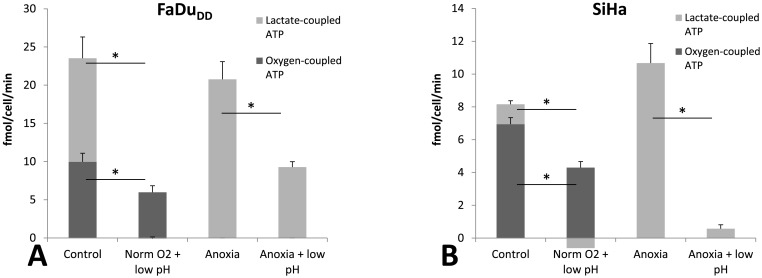
Energy metabolism following acidosis. The cellular ATP budget was calculated from OCR and LPR as detailed in Methods and Materials. The total bar height represents total ATP production, which consists of the sum of ATP generated from OXPHOS (dark gray) and ATP generated from glycolysis (light gray). The data is the mean values from three to four independent experiments (+/- SEM). (*) indicates p values <0.05 between the indicated treatments.

To assess whether the reduction in protein synthesis rate is part of an overall metabolic rate depression, lactate production rate (LPR) and oxygen consumption rate (OCR) was measured in cells exposed to either anoxia, low pH or a combination of both. In oxygenated tumor cells acidosis caused highly significant decreases in LPR (in fmol/cell/min) from 13.6±3.8 to -0.03±0.30 (P = 0.02) and from 1.2±0.3 to -0.6±0.6 (P = 0.01) in FaDu_DD_ and SiHa cells, respectively, suggesting that acidosis minimizes or even reverts the lactate flux (Fig 5). Similarly, the transition from pH 7.5 to 6.3 caused decreases in OCR (in fmol/cell/min) from 2.3±0.3 to 1.3±0.3 (P = 0.07) and from 1.5±0.03 to 0.9±0.08 (P = 0.003) in FaDu_DD_ and SiHa cells, respectively. As expected, anoxia stimulated anaerobic glycolysis, especially in SiHa which is consistent with previous observations [20]. Under anoxic conditions, LPR dropped significantly from 20.8±2.3 to 9.3±0.7 in FaDu_DD_ (P = 0.01) and from 10.7±1.2 to 0.6±0.2 (P = 0.001) in SiHa when pH was lowered from 7.5 to 6.3. From these data sets, the number of ATP equivalents generated in glycolysis from the conversion of glucose to lactate (lactate-coupled) and oxidative phosphorylation (OXPHOS), respectively, were calculated as described in materials and methods. This basal energy budget is depicted in Fig 5. It is evident that low pH induces an overall metabolic rate depression (the sum of glycolytic and OXPHOS generated ATP), and shifts the cells towards a non-glycolytic phenotype. As expected, anoxia caused a compensatory increase in glycolytic flux (Pasteur effect), but for SiHa the combination of anoxia and low pH suppressed overall metabolic rate dramatically.

## Discussion

The tumor microenvironment is characterized by hypoxia, low pH and high lactate. Hypoxia stimulates anaerobic energy production and the formation of lactic acid as a compensation for reduced mitochondrial ATP production (Pasteur effect), but high intrinsic glycolytic dependency (Warburg effect) weakens the Pasteur effect in tumor cells and thus the spatial link between low pH and hypoxia, in the sense that areas with low pH despite of normoxic conditions exists. It is therefore important to distinguish between the phenotypes of cells exposed to either or both, hypoxia and acidosis. It has previously been observed for a number of genes, that gene expression during hypoxia can be affected by low extracellular pH [12–14,22]. In a previous in vitro study the gene expression patterns of squamous cell carcinoma cell lines under hypoxia and/or low extracellular pH was studied. Based on these data, 29 genes characterized by being upregulated under hypoxic conditions and furthermore being independent of pH fluctuations were identified as potential classifier genes across a range of cell lines [18]. These genes have been evaluated in a clinical material, which has led to the development of a 15-gene hypoxia classifier, which attain both prognostic and predictive impact for hypoxic modification in head and neck cancer [23].

The cellular effects of acidosis has been evaluated in a range of studies in connection to ischaemia, which is known to reduce pH in local tissue. In some of the studies an effect on protein translation rate as a function of low pH has been shown [24,25]. In the present study we have both investigated the combined effect of hypoxia and acidosis, and sought to unravel the responses caused by the two tumor microenvironmental factors. We have identified a group of genes, which are induced by acidosis—independent of hypoxia. Several of the genes that were identified as upregulated by severe acidosis in a number of cell lines are involved in the translation process. Eukaryotic translation initiation factor 4A2, *EIF4A2*, is involved in the initiation phase of translation, which is required for the assembly of a complete ribosome at the start codon of the mRNA. Ribosomal protein L37, *RPL37*, encodes a ribosomal protein that is a component of the large 60S ribosomal subunit. JOSD3 (TATA box binding protein (TBP)-associated factor, RNA polymerase I, D, 41kDa (TAF1D)) plays a role in transcription of RNA polymerase I, which synthesizes pre-rRNA. Other genes identified to be upregulated during acidosis were involved in process such as transcription and signal transduction. Other studies have identified acidosis-induced genes [12,26,27], but there is however no overlap with the genes identified in the present study. This could be due to the analysis in the present study aiming at identifying pH induced genes not affected by hypoxia. In this study cells were treated with hypoxia and/or acidosis for 24 hours. Previous data has shown hypoxia induced gene expression to be affected after short term hypoxia (down to 3 hours) [14], pointing towards fluctuation in hypoxic level, and hence fluctuations in pH, to affect the gene expression patterns.

Since many of the genes we identified are involved in gene translation, we analyzed the effect of acidosis on the level of protein synthesis. Down regulation of protein synthesis have previously been reported as an effect of hypoxia [28–30], and is thought to be a mechanism to preserve energy and protect against the stress of hypoxia. In our study a combination of acidosis and hypoxia affected the cells to a much higher extent than any of the factors alone, and led to an almost complete shutdown of protein synthesis. Experimental data has shown acidosis (pH 6.4) in HK-2 cells to inhibit genes involved in protein translation [31]. The reason we find increased expression of genes associated with translation, even though translation is shown to be down-regulated, may be due to a feedback mechanism of the translation machinery. A previous in vitro study has demonstrated low pH to protect against oxygen–glucose deprivation induced cellular injury [31]. The proteins that remain to be translated during hypoxia and acidosis may be involved in mechanisms for cell survival.

Protein synthesis arrest was occurring simultaneously to an overall ATP- sparing metabolic rate depression (Fig 5). This supports that cells under simultaneous acidosis and hypoxia are responding with a different mechanism, than cells under hypoxia alone, which is also shown by the different gene expression patters that cells express in vitro depending on whether they are exposed to hypoxia alone, or hypoxia together with acidosis [12,18].

Whereas hypoxia force cells towards a glycolytic phenotype acidosis has oppositely been shown to stimulate a transition to a more efficient energetic phenotype with a mitochondrial ATP synthesis, and under some conditions, lactate may even be taken up and oxidized [32]. Furthermore, acidosis can override the glycolytic change induced during mild hypoxia of 1% O_2_ and this may be an adaptive strategy to survive in an energy-restricted microenvironment [33]. During severe hypoxia, cells are forced to a glycolytic energy production (Fig 5), but the large decrease in macromolecule synthesis and total ATP requirement may serve as an adaptive response under such stressful conditions. The signal transduction pathways that fine tunes energy metabolism and metabolite fluxes under these opposing microenvironmental stresses are not fully understood, but interfering with them may make tumor cells vulnerable to energetic stress.

We demonstrate here that the influence of hypoxia and acidosis causes different responses, both in gene expression and in de novo protein synthesis, depending on whether the two factors induced alone or overlapping, and as such it is important for in vivo studies to take this into account.

## Supporting Information

S1 Fig Unsupervised hierarchical clustering of microarray data for FaDu_DD_ and SiHa cells (21, 5, 1, 0.1, 0.01 or 0% oxygen at pH 6.3 or 7.5). (PDF)

S2 Fig Multiclass Significance Analysis of Microarray (SAM). The top 25 of probe sets induced at low pH independent of oxygen concentration, at low oxygen independent at pH, and induced at low oxygen only at normal pH.
(PDF)

S3 Fig Data from Fig 2, analyzed within each pH. (PPTX)

S4 Fig Additional gels from pulse experiments. (PPTX)

S1 Methods Supplementary Methods. (DOCX)

S1 Table Array data: Fold difference of genes found to be differentially expressed by low pH. Blue columns indicates oxygen regulation, green columns indicates pH regulation.
(DOCX)
