# Supplementary material for: Simultaneous Hypoxia and Low Extracellular pH Suppress Overall Metabolic Rate and Protein Synthesis In Vitro
Source: PLoS One. 2015 Aug 14;10(8):e0134955. doi: 10.1371/journal.pone.0134955 (PMC4537201; doi:10.1371/journal.pone.0134955)
Supplement: S1 Fig — (PDF) [file pone.0134955.s001.pdf]

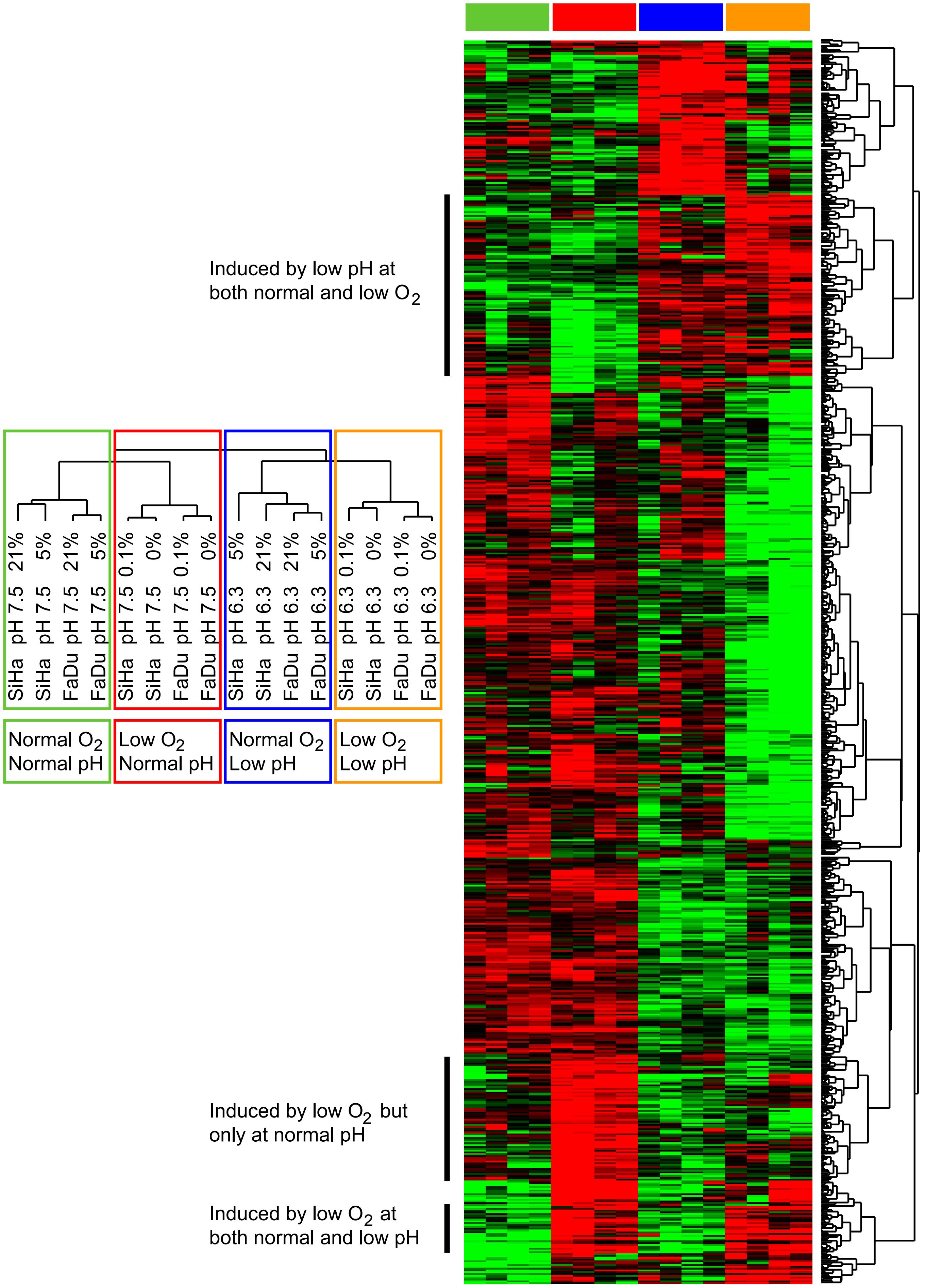

Supplementary figure S1
