# Supplementary material for: Simultaneous Hypoxia and Low Extracellular pH Suppress Overall Metabolic Rate and Protein Synthesis In Vitro
Source: PLoS One. 2015 Aug 14;10(8):e0134955. doi: 10.1371/journal.pone.0134955 (PMC4537201; doi:10.1371/journal.pone.0134955)
Supplement: S2 Fig — The top 25 of probe sets induced at low pH independent of oxygen concentration, at low oxygen independent at pH, and induced at low oxygen only at normal pH. (PDF) [file pone.0134955.s002.pdf]

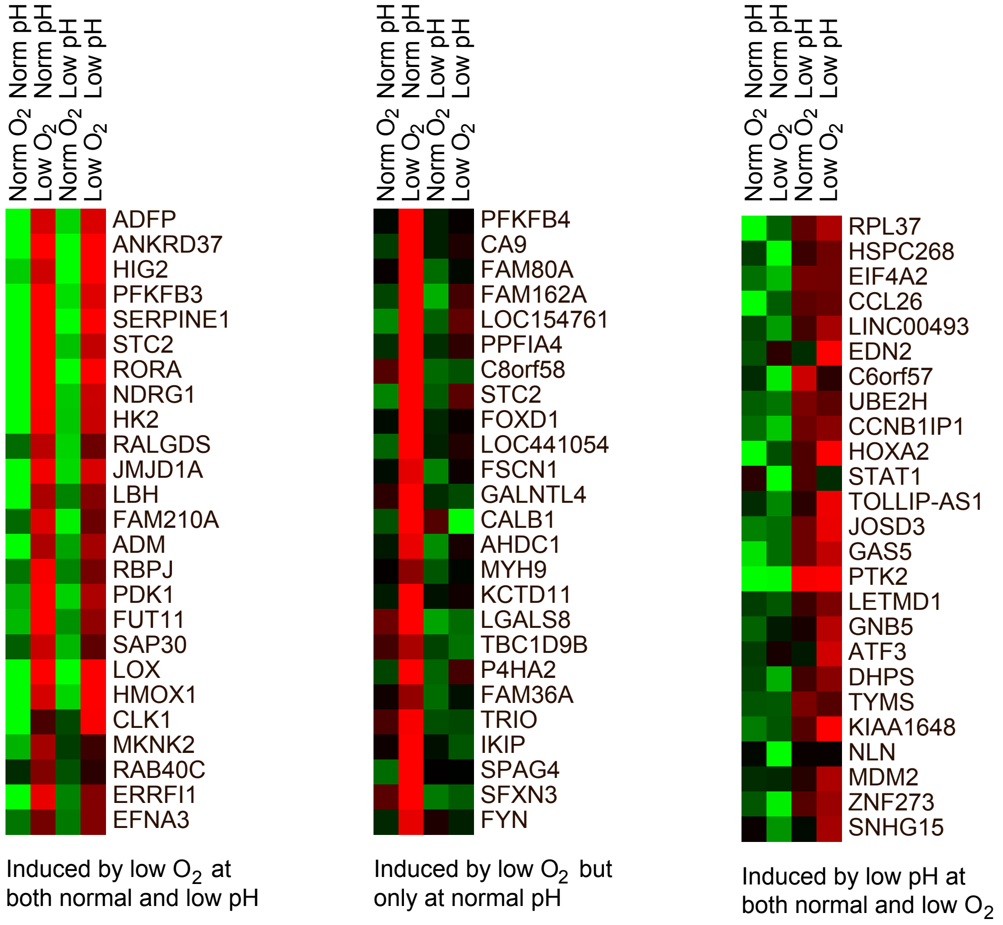

Supplementary figure S2
