# Supplementary material for: Simultaneous Hypoxia and Low Extracellular pH Suppress Overall Metabolic Rate and Protein Synthesis In Vitro
Source: PLoS One. 2015 Aug 14;10(8):e0134955. doi: 10.1371/journal.pone.0134955 (PMC4537201; doi:10.1371/journal.pone.0134955)
Supplement: S3 Fig — (PPTX) [file pone.0134955.s003.pptx]

## Slide 1
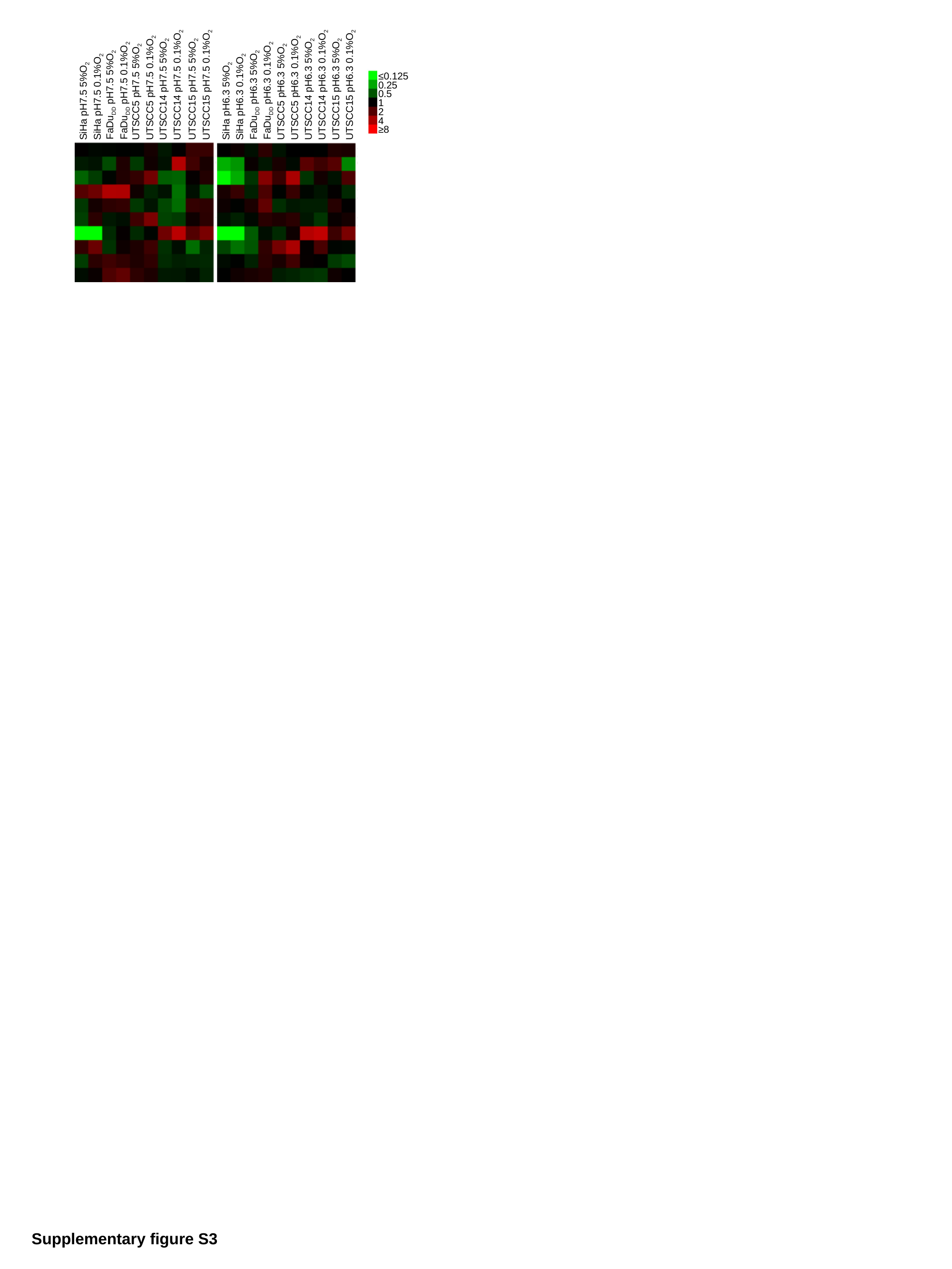

≤0.125
0.25
0.5
1
2
4
≥8
UTSCC14 pH7.5 0.1%O2
UTSCC15 pH7.5 0.1%O2
UTSCC14 pH6.3 0.1%O2
UTSCC15 pH6.3 0.1%O2
UTSCC5 pH7.5 0.1%O2
UTSCC5 pH6.3 0.1%O2
UTSCC14 pH7.5 5%O2
UTSCC15 pH7.5 5%O2
UTSCC14 pH6.3 5%O2
UTSCC15 pH6.3 5%O2
FaDuDD pH7.5 0.1%O2
FaDuDD pH6.3 0.1%O2
UTSCC5 pH7.5 5%O2
UTSCC5 pH6.3 5%O2
FaDuDD pH7.5 5%O2
FaDuDD pH6.3 5%O2
SiHa pH7.5 0.1%O2
SiHa pH6.3 0.1%O2
SiHa pH7.5 5%O2
SiHa pH6.3 5%O2
Supplementary figure S3
