# Supplementary material for: Simultaneous Hypoxia and Low Extracellular pH Suppress Overall Metabolic Rate and Protein Synthesis In Vitro
Source: PLoS One. 2015 Aug 14;10(8):e0134955. doi: 10.1371/journal.pone.0134955 (PMC4537201; doi:10.1371/journal.pone.0134955)
Supplement: S4 Fig — (PPTX) [file pone.0134955.s004.pptx]

## Slide 1
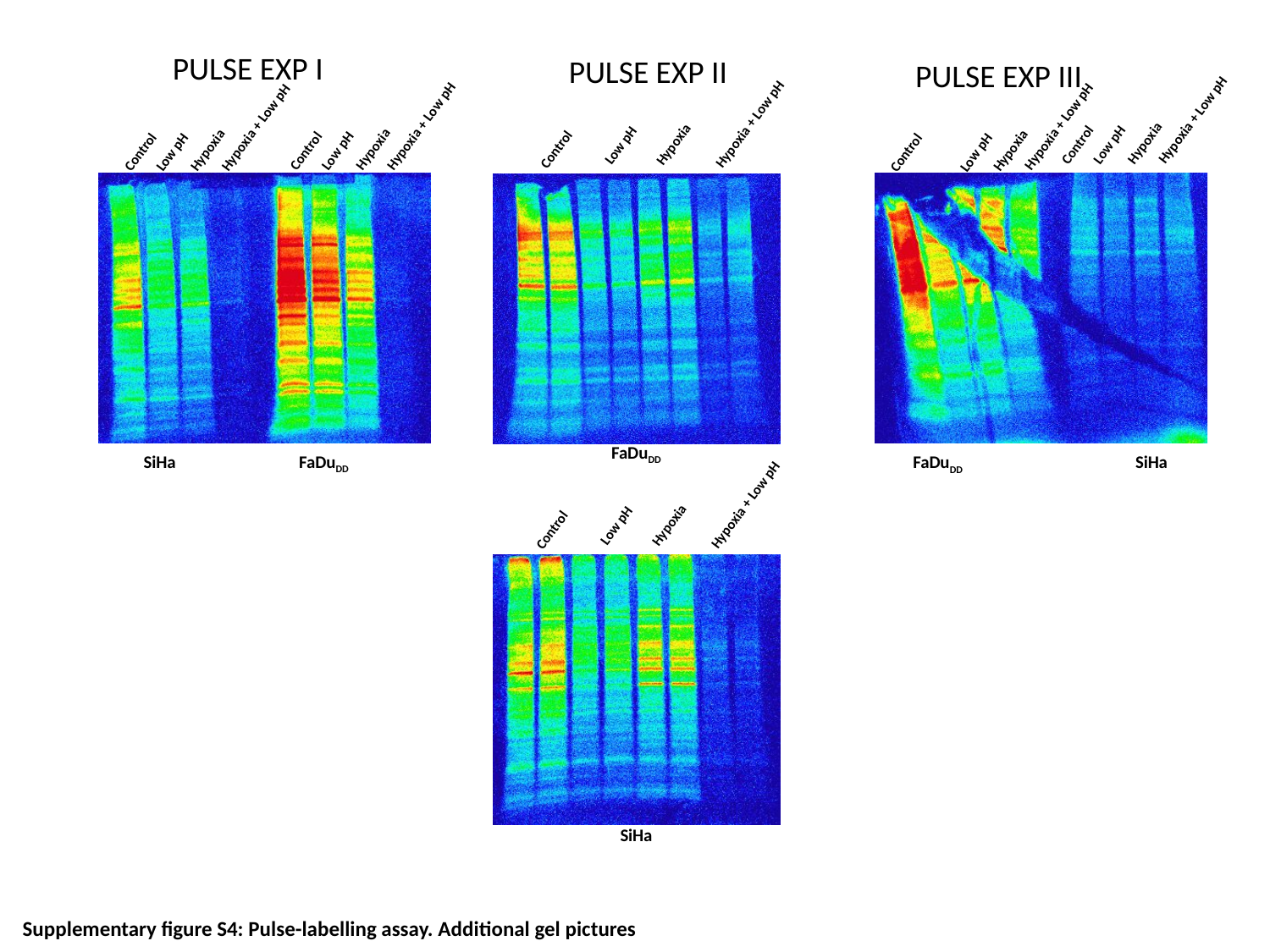

PULSE EXP I
PULSE EXP II
PULSE EXP III
Hypoxia + Low pH
Hypoxia
Low pH
Control
Hypoxia + Low pH
Hypoxia
Low pH
Control
Hypoxia + Low pH
Hypoxia
Low pH
Control
Hypoxia + Low pH
Hypoxia
Low pH
Control
Hypoxia + Low pH
Hypoxia
Low pH
Control
FaDuDD
Hypoxia + Low pH
Hypoxia
Low pH
Control
SiHa
FaDuDD
SiHa
FaDuDD
SiHa
Supplementary figure S4: Pulse-labelling assay. Additional gel pictures
