# Supplementary material for: Simultaneous Hypoxia and Low Extracellular pH Suppress Overall Metabolic Rate and Protein Synthesis In Vitro
Source: PLoS One. 2015 Aug 14;10(8):e0134955. doi: 10.1371/journal.pone.0134955 (PMC4537201; doi:10.1371/journal.pone.0134955)
Supplement: S1 Methods — (DOCX) [file pone.0134955.s005.docx]

**S1 Methods. Supplementary Methods**

The included microarray data for FaDu_DD_ and SiHa cells were part of a larger study, were the following conditions were investigated: 21, 5, 1, 0.1, 0.01 or 0% oxygen at pH 6.3 or 7.5.

The following analysis was performed for the FaDu_DD_ and SiHa data only:

Data was analyzed by unsupervised hierarchical clustering (figure S1). This demonstrated that the genes separated into main groups representing the cell lines. Within each cell line, the samples then clustered into high and low pH, showing that acidosis affected cellular gene expression profiles more profoundly than hypoxia.

Using a multiclass Significance Analysis of Microarray (SAM) of probesets expressed above the 25% quartile in more than half of the samples, we selected 1036 probesets (false discovery rate ~1%) that distinguished between four groups in SiHa and FaDu_DD_. The four groups were defined as for further analysis, combining both cell lines, **“**Normal oxygen, normal pH” (21%, pH 7.5; 5%, pH 7.5), “Low oxygen, normal pH” (0.1%, pH 7.5; 0%, pH 7.5;), “Normal oxygen, low pH” (21%, pH 6.3; 5%, pH 6.3), “Low oxygen, low pH” (0.1%, pH 6.3; 0%, pH 6.3). A hierarchical clustering (median centered genes, complete linkage) of samples based on the selected probe sets, sorted the genes in clusters. This made it possible to select a set of probe sets induced at low pH independent of oxygen concentration; namely probe sets upregulated in the two sample groups “Normal oxygen, low pH” and “Low oxygen, low pH”. It was also possible to select a fraction of probe sets induced at low oxygen only at normal pH, this was the probe sets upregulated only in the sample group “Low oxygen, normal pH”. Finally, we could select probe sets induced at low oxygen independent at pH. This group consisted of probe sets upregulated in both the two sample groups “Low oxygen, normal pH” and “Low oxygen, low pH”. For each of these fractions of probe sets, the genes were sorted and represented based on SAM score. The top 25 for each fraction is shown on figure S2.
