# Supplementary material for: Simultaneous Hypoxia and Low Extracellular pH Suppress Overall Metabolic Rate and Protein Synthesis In Vitro
Source: PLoS One. 2015 Aug 14;10(8):e0134955. doi: 10.1371/journal.pone.0134955 (PMC4537201; doi:10.1371/journal.pone.0134955)
Supplement: S1 Table — Blue columns indicates oxygen regulation, green columns indicates pH regulation. (DOCX) [file pone.0134955.s006.docx]

|  | **SiHa** | | | | | | **FaDu** | | | | | | **UTSCC5** | | | | | |
| --- | --- | --- | --- | --- | --- | --- | --- | --- | --- | --- | --- | --- | --- | --- | --- | --- | --- | --- |
| **Gene** | **0.1% norm pH > 5% norm pH - Level** | **0.1% low pH > 5% low pH - Level** | **0.1% low pH > 5% norm pH - Level** | **5% low pH > 5% norm pH - Level** | **0.1% low pH > 5% norm pH -Level** | **0.1% low pH > 0.1% norm pH -Level** | **0.1% norm pH > 5% norm pH - Level** | **0.1% low pH > 5% low pH - Level** | **0.1% low pH > 5% norm pH - Level** | **5% low pH > 5% norm pH - Level** | **0.1% low pH > 5% norm pH -Level** | **0.1% low pH > 0.1% norm pH -Level** | **0.1% norm pH > 5% norm pH - Level** | **0.1% low pH > 5% low pH - Level** | **0.1% low pH > 5% norm pH - Level** | **5% low pH > 5% norm pH - Level** | **0.1% low pH > 5% norm pH -Level** | **0.1% low pH > 0.1% norm pH -Level** |
| **EIF4A2** | 0.92 | 0.89 | 2.78 | 2.48 | 2.78 | 3.01 | 1.03 | 0.61 | 3.82 | 2.33 | 3.82 | 3.72 | 1.23 | 0.86 | 2.60 | 2.23 | 2.60 | 2.11 |
| **GAS5** | 1.85 | 1.14 | 4.88 | 5.58 | 4.88 | 2.63 | 1.05 | 0.56 | 4.92 | 2.76 | 4.92 | 4.67 | 1.34 | 0.83 | 4.14 | 3.45 | 4.14 | 3.10 |
| **IFNGR2** | 1.21 | 0.86 | 4.06 | 3.51 | 4.06 | 3.35 | 1.17 | 0.94 | 2.24 | 2.09 | 2.24 | 1.91 | 0.87 | 1.06 | 1.64 | 1.74 | 1.64 | 1.89 |
| **IL1RAP** | 1.38 | 0.54 | 2.39 | 1.29 | 2.39 | 1.73 | 1.36 | 0.22 | 13.46 | 2.98 | 13.46 | 9.87 | 1.69 | 0.39 | 11.62 | 4.52 | 11.62 | 6.88 |
| **IRF6** | 0.98 | 1.15 | 2.23 | 2.57 | 2.23 | 2.27 | 1.56 | 0.58 | 4.50 | 2.63 | 4.50 | 2.89 | 1.34 | 0.71 | 5.34 | 3.82 | 5.34 | 3.98 |
| **JOSD3** | 2.31 | 0.93 | 5.36 | 4.97 | 5.36 | 2.32 | 0.90 | 0.53 | 2.86 | 1.53 | 2.86 | 3.19 | 1.15 | 0.73 | 4.39 | 3.19 | 4.39 | 3.82 |
| **KLHL7** | 1.23 | 0.78 | 3.22 | 2.52 | 3.22 | 2.62 | 0.98 | 0.42 | 1.81 | 0.75 | 1.81 | 1.84 | 0.66 | 0.61 | 5.73 | 3.52 | 5.73 | 8.72 |
| **OSMR** | 1.58 | 1.47 | 1.25 | 1.84 | 1.25 | 0.79 | 1.69 | 0.34 | 10.74 | 3.61 | 10.74 | 6.35 | 1.30 | 0.65 | 15.21 | 9.82 | 15.21 | 11.73 |
| **RPL37** | 2.38 | 1.15 | 4.82 | 5.55 | 4.82 | 2.03 | 1.07 | 0.66 | 6.41 | 4.23 | 6.41 | 5.98 | 1.67 | 0.90 | 3.22 | 2.90 | 3.22 | 1.93 |
| **TXNIP** | 1.05 | 0.80 | 3.67 | 2.94 | 3.67 | 3.48 | 2.37 | 1.30 | 15.54 | 20.26 | 15.54 | 6.57 | 1.84 | 1.32 | 14.96 | 19.81 | 14.96 | 8.15 |
|  | **UTSCC14** | | | | | | **UTSCC15** | | | | | |  |  |  |  |  |  |
| **Gene** | **0.1% norm pH > 5% norm pH - Level** | **0.1% low pH > 5% low pH - Level** | **0.1% low pH > 5% norm pH - Level** | **5% low pH > 5% norm pH - Level** | **0.1% low pH > 5% norm pH -Level** | **0.1% low pH > 0.1% norm pH -Level** | **0.1% norm pH > 5% norm pH - Level** | **0.1% low pH > 5% low pH - Level** | **0.1% low pH > 5% norm pH - Level** | **5% low pH > 5% norm pH - Level** | **0.1% low pH > 5% norm pH -Level** | **0.1% low pH > 0.1% norm pH -Level** |  |  |  |  |  |  |
| **EIF4A2** | 1.25 | 1.01 | 3.03 | 3.04 | 3.03 | 2.43 | 1.00 | 1.04 | 2.00 | 2.08 | 2.00 | 1.99 |  |  |  |  |  |  |
| **GAS5** | 0.74 | 1.01 | 4.46 | 4.49 | 4.46 | 6.07 | 0.95 | 1.34 | 2.13 | 2.85 | 2.13 | 2.24 |  |  |  |  |  |  |
| **IFNGR2** | 1.01 | 1.05 | 2.51 | 2.63 | 2.51 | 2.49 | 0.83 | 1.12 | 3.52 | 3.94 | 3.52 | 4.23 |  |  |  |  |  |  |
| **IL1RAP** | 0.96 | 0.57 | 10.84 | 6.15 | 10.84 | 11.32 | 1.26 | 0.46 | 7.74 | 3.57 | 7.74 | 6.13 |  |  |  |  |  |  |
| **IRF6** | 1.42 | 0.92 | 7.54 | 6.97 | 7.54 | 5.32 | 1.28 | 0.72 | 4.33 | 3.11 | 4.33 | 3.39 |  |  |  |  |  |  |
| **JOSD3** | 1.10 | 1.04 | 4.79 | 4.96 | 4.79 | 4.34 | 0.96 | 1.15 | 2.42 | 2.77 | 2.42 | 2.51 |  |  |  |  |  |  |
| **KLHL7** | 0.45 | 1.12 | 4.03 | 4.50 | 4.03 | 8.91 | 0.60 | 1.43 | 3.36 | 4.81 | 3.36 | 5.62 |  |  |  |  |  |  |
| **OSMR** | 1.31 | 0.58 | 12.79 | 7.41 | 12.79 | 9.74 | 1.83 | 1.02 | 11.12 | 11.32 | 11.12 | 6.06 |  |  |  |  |  |  |
| **RPL37** | 1.06 | 1.29 | 4.15 | 5.35 | 4.15 | 3.91 | 1.26 | 0.91 | 3.93 | 3.57 | 3.93 | 3.13 |  |  |  |  |  |  |
| **TXNIP** | 4.98 | 1.26 | 19.16 | 24.17 | 19.16 | 3.84 | 0.72 | 6.00 | 2.00 | 12.01 | 2.00 | 2.77 |  |  |  |  |  |  |

Supplementary Table S1 Array data: Fold difference of genes found to be differentially expressed by low pH. Blue columns indicates oxygen regulation, green coloumns indicates pH regulation.
